# Management of Upper Tract Urothelial Carcinoma

**DOI:** 10.1155/2009/492462

**Published:** 2009-02-05

**Authors:** Norm D. Smith

**Affiliations:** The Robert H. Lurie Comprehensive Cancer Center and Department of Urology, Feinberg School of Medicine, Northwestern University, Chicago, IL 60611-3008, USA

Upper tract urothelial carcinoma (UTUC) of the kidney and ureter is a rare neoplasm with roughly 3000 cases diagnosed in the United States in 2007, compared to approximately 67000 cases of bladder cancer. UTUC accounts for approximately 10% of cancers arising from the kidney and less than 5% of all urothelial malignancies. Predominantly due to the rarity of the disease, there is only limited data to guide clinicians in decision making which consists mostly of small retrospective studies and expert opinion. As such, this special issue of Advances in Urology “Comprehensive Management of Upper Tract Urothelial Carcinoma” focuses both on controversies in management as well as practical issues in the care of patients with UTUC. As the Guest Editor, my goal has been to ask a series of questions and seek answers from international experts in the field.

The first practical matter is to address when endoscopic management of UTUC is appropriate? Our first article “Endoscopic Management of Upper Tract Urothelial Carcinoma” by Katie Moore et al. addresses this issue generally. Then, the paper entitled “Endourologic Management of Upper Tract Transitional Cell Carcinoma Following Cystectomy and Urinary Diversion” by Jeffrey John Tomaszewski et al. tackles the difficult situation of UTUC after radical cystectomy and urinary diversion. Finally, after initial endoscopic management of UTUC, what is the role of upper tract topical chemotherapy or BCG? This topic is the subject of the paper entitled “Review of Topical Treatment of Upper Tract Urothelial Carcinoma” by Kenneth G. Nepple et al.

The next issue of focus is the clinical scenario when UTUC cannot be managed endoscopically. Are laparoscopic and open nephroureterectomy equivalent operations? What is the best way to handle the distal ureter and bladder cuff? These challenging topics are addressed by a series of articles: “Laparoscopic Nephroureterectomy: Oncologic Outcomes and Management of Distal Ureter: Review of the Literature” by Andre Berger and Amr Fergany, “Laparoscopic Nephroureterectomy: The Distal Ureteral Dilemma” by Shalom Srirangam et al., as well as “Laparoscopic Nephroureterectomy and Management of the Distal Ureter: A Review of Current Techniques and Outcomes” by Davis Viprakasit et al. Regardless of open versus laparoscopic approaches to nephroureterectomy, what is the role and extent of lymphadenectomy in the management of UTUC? This critical issue is dealt with in the paper entitled “Retroperitoneal Lymph Nodes in Transitional Cell Carcinoma of the Kidney and Ureter” by Shilajit D. Kundu and Scott E. Eggener.

The last matter is the management of patients with locally advanced or metastatic UTUC. Is there a role for adjuvant chemotherapy? What are the best agents for metastatic UTUC? Can data from bladder cancer be applied to the upper tracts? These timely topics are concentrated upon in the article “The Role of Chemotherapy in Upper Tract Urothelial Carcinoma” by Peter H ODonnell and Walter M. Stadler. Finally, these various issues are brought together and placed into perspective in the review entitled “Comprehensive Management of Upper Tract Urothelial Carcinoma” by Georgios Koukourakis et al.

It has been my privilege to serve as the Guest Editor for this special issue of Advances in Urology “Comprehensive Management of Upper Tract Urothelial Carcinoma.”

*Norm D. Smith*

